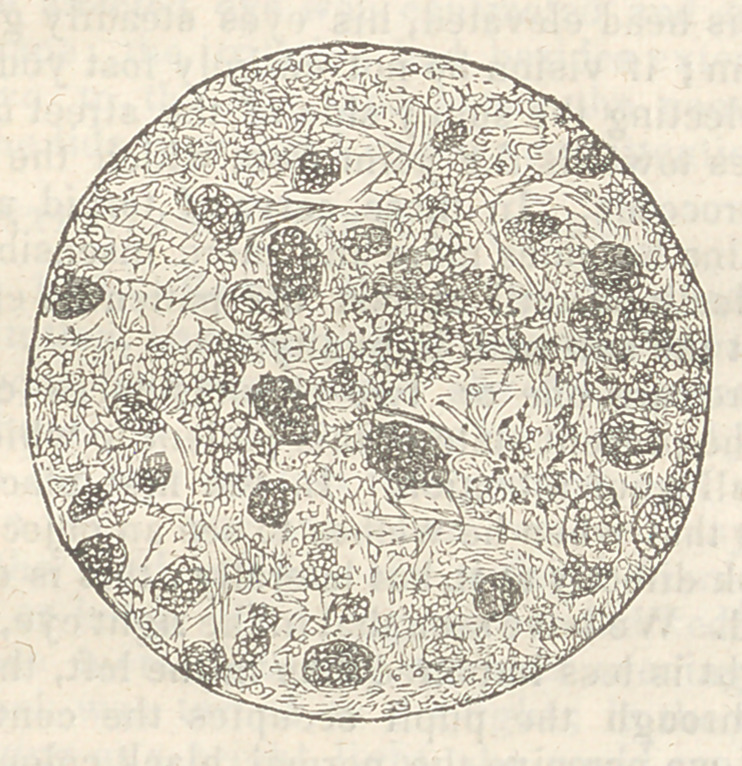# Clinic of Professor Mütter

**Published:** 1843-12-23

**Authors:** H. T. Child


// THE MEDICAL EXAMINER,

**And Retrospect of the Medical Sciences.**

Vol. VI.] PHILADELPHIA, SATURDAY, DECEMBER 23, 1843. [No. 25.

### CLINICAL LECTURES AND REPORTS.

### JEFFERSON MEDICAL COLLEGE.

CLINIC OF PROFESSOR MÜTTER.

(Reported by H. T. Child.*)

CASE I.

TUMOUR OF THE NAPE OF THE NECK.

*November 28th*, 1843.—Professor Pancoast introduced a female patient with a tumour on the nape of the neck, of thirteen years standing, which he proceeded to remove by carefully dissecting out the whole sac. It was an encysted, steatomatous tumour, about one and a half inches in diameter. A small artery required a ligature, and the parts were then drawn together by means of adhesive straps, and a light dressing applied over the whole.

*Microscopic examination.*—The contents of this tumour contain numerous beautiful rhomboidal crystals, most of which are very regular, and all seem perfectly defined. Whether other tumours of this kind contain these crystals I have not yet had means of ascertaining: if so, this is a fact which appears to have been overlooked by previous observers. The form of the crystals is seen in the figure, which represents the field of the microscope under a magnifying power of about 600 diameters. The crystals are lamellar, and very thin, not exceeding in thickness the thirty thousandth of an inch. They seem not to be soluble in water, slowly soluble in alcohol, and are readily dissolved in oil of turpentine. They remain unchanged, floating in nitric or muriatic acid diluted with an equal quantity of water, and are equally unaffected by a strong solution of caustic potash, which dissolves all but the crystals.

Professor Mütter then introduced

CASE II.

CONGENITAL ICTHYOSIS.

M. A——, a female, aged 10 years. Lymphatic temperament; general health good.

This child you may recollect, gentlemen, has already been presented at the clinic; but I bring her before you to-day in order to exhibit the happy influence of the treatment prescribed. The skin, you observe, is becoming pale and smooth; the scabs, which covered her literally from head to foot, are falling off, leaving a smooth and healthy surface, where before there was nothing but a loathsome and fetid ulcer. Her cheeks are becoming rosy, her tongue clean, her appetite excellent, and her excretions regular. Her hands and feet,* although distorted, look a little more natural, and the joints are more flexible.

The remedies, from the employment of which so much benefit has been derived, are general mucilaginous baths every other day, weak sulphate of copper washes to the ulcers, (gr. j. to ℥ij. of water,) a good diet, warm clothing, fresh air, and, above all, the internal use of "*Donovan's Solution*," the Liquor Hydriodatis Arsenici et Hydrargyri. I cannot too strongly recommend this article in certain forms of cutaneous disease; those, for instance, in which a steady and protracted alterative course is required, and where the stomach is too irritable to bear either iodine, arsenic, or mercury, in their usual forms of administration. You have seen its good effects in the cases of lupus and some other diseases; but in none has its beneficial influence been so marked as in the case before us. We shall continue the treatment. We began with three drops three times a day, and have gradually increased, till at present she takes nine drops three times a day.

CASE III.

ORIGINALLY A CASE OF LUPUS OF THE LOWER LID,

For which an operation was performed last winter. See Medical Examiner for Feb. 14, 1843, No. 2, Vol. vi.

* The regular reports commence with this lecture. Since Professor Mütter's service commenced on the 4th of October, he has operated for cataract—removed a glandular tumour—performed two operations for hare-lip, with fissure of the hard and soft palates in infants—an extensive plastic operation for contraction of the fingers and thumb—extirpated two tonsils—operated for fistula lacrymalis—catheterised the Eustachian tube, &c. Professor Pancoast has also introduced from his private practice a number of patients, in order that the class might have an opportunity of witnessing the operations. Of these, there was a case of amputation of the left mamma—an operation for cancer of the lip—and a case of paracentesis cerebri.

* The fingers and toes of this child are webbed; and the adhesions so close and perfect, that in the feet and left hand especially it is almost impossible to distinguish the natural lines of separation for the toes and fingers.

Many of you, I have no doubt, recollect Mrs. T——, upon whom, during the last season, I performed a most extensive *Blepheroplastic* operation for the cure of an obstinate noli me tangere. The operation has proved, as you perceive, eminently successful; not a vestige of the disease, from which she had suffered severely for seven years, now remains: and it is really almost impossible, without a minute examination, to say upon which eye it was performed. This case is highly interesting, strongly substantiating, as it does, the views entertained by Martinet de la Creuse, Deiffenbach, Phillips and others, in relation to the influence of the application of healthy tissue to a part from which cancerous formations have been previously removed.

In this and many other cases, I have unquestionably accomplished a cure by performing a plastic operation at the time the diseased tissue was removed, instead of allowing the parts to heal by granulation.

I do not pretend to explain the fact, *causa latat vis est notissima;* but certain I am, that no better advice can be given you than this: *endeavour, whenever you operate for cancerous or malignant disease, to cover in the surface from which you remove it with healthy skin;* and rest assured that, by so doing, you will very much diminish the chances of a reappearance of the affection.

### CASE IV.

### ORIGINALLY A CASE OF CANCER OF THE LIP.

Here is another highly interesting case, and one that fully bears me out in what I have just been telling you. Wm. L——, some of you will recollect, submitted during the last winter to a very serious and extensive *Cheiloplastic* operation, for the removal of cancer of the lower lip, involving the whole organ. This diagram explains to you the operation I performed; and although no single operation will answer in every case, yet where this can be performed, I vastly prefer it to any other method. It consists in first removing the diseased tissue by a semilunar incision, carried below it and down to the bone. Then, in order to obtain a proper flap for the lip, carrying a perpendicular incision directly through the centre of the chin, and as far down the neck as may be necessary; next commencing at the terminal extremity of this incision, carry another, curvilinear in shape, parallel with that made for the removal of the tumour, along the base of the jaw or lower, as the case may be, until it reaches a point opposite the commissure of the mouth. This accomplished, dissect up the flap included between the two incisions, and then perform a similar operation on the opposite side. The two flaps being thus prepared, are to be lifted up into the position originally occupied by the lip, and united to each other at the mesial line by the twisted suture. This operation, gentlemen, I believe belongs to me; for I performed it as long since as 1834. Mr. Buchanan, of Glasgow, has recently published a paper upon this subject, and supposes the method to have originated with himself; but, as I have just told you, I performed a similar operation several years since. The operation of Dupuytren, which consists in simply removing the diseased mass by a curvilinear incision, and then trusting to granulations to fill up the cavity, is rarely successful, and the disease, so far as my experience goes, is more apt to return than when plastic surgery is had recourse to.

Nor is the operation in which a flap is detached from the chin and throat by a single perpendicular incision on each side, and then forcibly dragged upwards and retained in its place by ligatures, more to be relied on. A few successful cases, among them that of Mr. Earle, published in the 12th volume of the Medico-Chirurgical Transactions, are to be met with, but they are not sufficiently numerous to induce us to prefer the operation to that just described.

Of course, the simple V incision can only be practised when the tumour is small; but where the extent of the disease does not contraindicate its employment, it will answer an excellent purpose.

### CASE V.

### CEPHALOMATOUS TUMOUR.

A. L——, æt. 50 years, has a cephalomatous tumour of the lower lid, involving the entire organ, and projecting into the orbit at the inner canthus. In size, externally, it equalled a hen's egg, was livid in colour, with here and there an ulcerous spot of a lighter hue, and presented the consistence of the medullary tissue of a healthy brain. Its attachments were confined to the inner canthus—all the other portions of the tumour being moveable. The general health of the patient is excellent, and the lymphatics in the vicinity not at all enlarged or diseased. There is some shooting pain in the tumour, and it increases rapidly in size. The success attending the employment of plastic surgery in similar cases, induced Professor Mütter to perform the following operation, which differs in some respects from that employed in the case of Mrs. T—— and others.

The hair having been previously shaved from the temple and cheek, the patient was seated with his head resting against the chest of an assistant, and the operation commenced by carrying an oblique incision from the outer canthus down to the centre of the cheek; another was then made from the inner canthus to the terminal extremity of the first. The tumour was thus included between the two, and then immediately dissected out as completely as possible, leaving a space of the shape of the letter V. After its removal, the disease was found to extend deep into the orbit, and also to involve a portion of the upper lid. To get rid of this, the diseased portion of the lid was at once cut away, and then the base of the tumour seized with a pair of double hooked forceps, brought forward as much as possible, and carefully separated with the scalpel from all its attachments. This part of the operation was very painful, as the tumour adhered closely to the ball of the eye, which it had displaced from its natural position. After the removal of the diseased mass, both the ball of the eye and the adjacent bones of the orbit were found perfectly healthy. The patient was allowed to rest a few minutes, and the bleeding having in a great measure ceased, the next step of the operation was commenced. Starting from the superior extremity of the first incision at the external canthus, the scalpel was carried upwards about an inch above the orbit, and towards the temple, then turned back, so as to mark out a circular flap about three quarters of an inch in length, from the base of which it was carried outwards, in a *curvilinear* direction, for about an inch and a quarter, in order that this margin might correspond with the natural curve of the eyelid. From the terminal extremity of this, another incision was carried downwards and forwards until it reached a point opposite the union of the two first. The flap included in these incisions was then dissected up, brought over the raw surface from which the diseased tissue had been removed, and attached to the skin along the nose by several stitches of the interrupted suture; the round portion at the upper margin fitting accurately the space left in the upper lid by the removal of its diseased tissue. The surface from which the flap had been removed was then partly closed by sutures, and the other portions left to heal by granuations, and dressed with water dressing. The treatment ordered consisted in the application of *cool water to the flap*, maintaining the head in an elevated position, and placing the patient upon the antiphlogistic system.

Professor M. observed that there were many other operations proposed for the restoration of the eyelids, most of which would be found either described or referred to in the report of his lecture on Blepheroplastic operations, published in the Medical Examiner for February 14, 1843, No. 2, Vol. vi.

*Microscopic Examination of the Tumour.*—The accompanying drawing represents a thin slice of the medullary part of the tumour magnified about 600 diameters. In this were seen no bloodvessels, and very few fibres of cellular tissue. The hematoid portion contained many globules like those seen in the figure, within which are nuclei or granules; and besides numerous bloodvessels, this reticulated tissue was supplied with many tubes, larger than the minute bloodvessels, which tubes terminated in a bulbous extremity filled with blood globules. Some of the drawings of Müller compare quite well with the above.

[NOTE.—The examination of the tumors was made by Mr. Southwick, a member of the class, who, with a laudable zeal, is devoting much time to microscopic investigations.—T. D. M.]

## Figures and Tables

**Figure f1:**
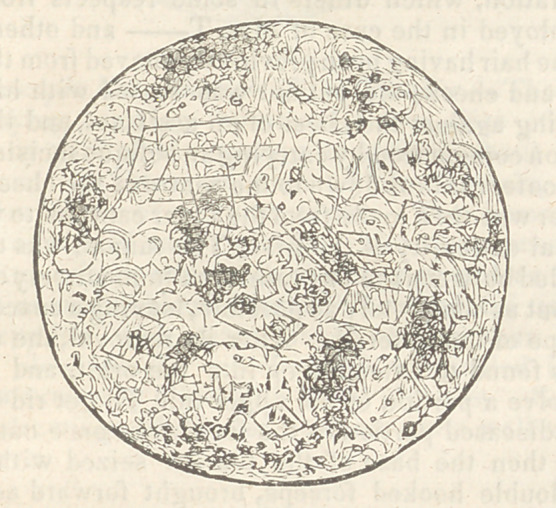


**Figure f2:**